# Genetic Dissection of Sympatric Populations of Brown Planthopper, *Nilaparvata lugens* (Stål), Using DALP-PCR Molecular Markers

**DOI:** 10.1100/2012/586831

**Published:** 2012-04-19

**Authors:** M. A. Latif, M. Y. Rafii, M. S. Mazid, M. E. Ali, F. Ahmed, M. Y. Omar, S. G. Tan

**Affiliations:** ^1^Department of Crop Science, Faculty of Agriculture, Universiti Putra Malaysia, 43400, Serdang, Selangor, Malaysia; ^2^Plant Pathology Division, Bangladesh Rice Research Institute (BRRI), Gazipur 1701, Bangladesh; ^3^Institute of Tropical Agriculture, Universiti Putra Malaysia, 43400 UPM Serdang, Selangor, Malaysia; ^4^Institute of Nano Electronic Engineering (INNE), Universiti Malaysia Perlis, 01000 Kangar, Malaysia; ^5^Department of Biology, Faculty of Science, Universiti Putra Malaysia, 43400, Serdang, Selangor, Malaysia; ^6^Department of Cell and Molecular Biology, Faculty of Biotechnology and Molecular Science, Universiti Putra Malaysia, 43400, Serdang, Selangor, Bangladesh

## Abstract

Direct amplified length polymorphism (DALP) combines the advantages of a high-resolution fingerprint method and also characterizing the genetic polymorphisms. This molecular method was also found to be useful in brown planthopper, *Nilaparvata lugens* species complex for the analysis of genetic polymorphisms. A total of 11 populations of *Nilaparvata* spp. were collected from 6 locations from Malaysia. Two sympatric populations of brown planthopper, *N. lugens*, one from rice and the other from a weed grass (*Leersia hexandra*), were collected from each of five locations. *N. bakeri* was used as an out group. Three oligonucleotide primer pairs, DALP231/DALPR′5, DALP234/DALPR′5, and DALP235/DALPR′5 were applied in this study. The unweighted pair group method with arithmetic mean (UPGMA) dendrogram based on genetic distances for the 11 populations of *Nilaparvata* spp. revealed that populations belonging to the same species and the same host type clustered together irrespective of their geographical localities of capture. The populations of *N. lugens* formed into two distinct clusters, one was insects with high esterase activities usually captured from rice and the other was with low esterase activities usually captured from *L. hexandra. N. bakeri*, an out group, was the most isolated group. Analyses of principal components, molecular variance, and robustness also supported greatly to the findings of cluster analysis.

## 1. Introduction

The brown plant hopper, *Nilaparvata lugens* (Stål) (Homoptera: Delphacidae), is a major pest of rice, which is widely distributed from tropical to temperate areas of Asia and Australia. The insect is a phloem-feeder and is restricted to cultivated and wild rice as host plants. It causes “hopperburn” and complete wilting and drying of rice plants [1] and also transmits the grassy stunt and ragged stunt viral diseases [2]. Large-scale rice crop damage caused by the pest was reported in the 1970s in several South and Southeast Asian countries [1]. Another population of brown plant hopper was found to infest a weed grass, *Leersia hexandra*, which grows abundantly in canal near paddy fields in South East Asia [3, 4]. The weed infesting population of* N. lugens* fails to survive on rice plants. Conversely, rice infesting population of *N. lugens* does not thrive on grass [5]. Based on nymphal survival, virulence, ovipositional preference, mate choice, and hybridization experiments, Claridge et al. [5] suggested that the rice and *Leersia *infesting populations of brown planthopper (BPH) represented two distinct sympatric biological species. In recent studies, the analyses of isozymes and RAPD-PCR markers indicated that BPH with high esterase activity usually captured from rice plant, and those with low esterase activity usually captured from* L. hexandra* in Malaysia represent two distinct closely related sibling species [6, 7].

Direct amplification of length polymorphisms (DALPs) is a technique which uses arbitrarily primed PCR (AP-PCR) to produce genomic finger prints and to enable sequencing of DNA polymorphisms in any species. Oligonucleotide pairs were designed to produce a specific multibanded pattern for each individual of a population and between populations. This strategy combines the advantages of a high-resolution fingerprint technique and also characterizing the polymorphisms [8]. Higher number of polymorphic loci could be detected and isolated for sequencing in only one step. Therefore, this method is not simply another supplementary molecular fingerprinting technique but was designed from the very beginning to obtain nucleotide sequence information on DNA fragments from any genome with no need for a genomic library.

Genetic polymorphic markers, such as isozymes, RAPD and SSR, and nuclear or organelle DNA polymorphism, have been developed for a variety of studies on genetic diversity, population structures, and subdivisions [6, 7, 9–11]. The present study was undertaken to analyze genetic diversity as well as to detect genetic structures between two sympatric populations of *N. lugens*, one from rice and the other from weed grass, *L. hexandra*. We hypothesized that the molecular method newly applied in rice brown planthopper could be able to detect structures among the populations of brown planthopper species complex.

## 2. Experimental Section

### 2.1. Collection of Insect Populations

A total of 11 populations were collected from 6 locations. Two sympatric populations of* N. lugens*, one from rice and other from* L. hexandra*, were collected from each of five locations. The locations were Universiti Putra Malaysia (UPM), Tanjung Karang (TK), Melaka (MK), Perak (PK), and Sabah (SB). An out group, *N. bakeri* was also collected from Cameron Highlands (CH), Malaysia. Locations, host type, date of collection, population code are shown in Table 1. Each population consisted of twenty insects. All collected insects were frozen at −70°C for further use.

### 2.2. Esterase Activity Test

The individual insect used for DALP analysis was tested for esterase activity on a simple filter paper using the method reported by Pasteur and Georghiou [13].

### 2.3. DNA Extraction

DNA from individual insect was extracted by grinding single frozen adult insect with a glass rod in a 1.5 mL tube containing 20 *μ*L extraction buffer (0.1 M Nacl, 0.2 M Sucrose, 0.1 M Tris-HCL (pH 9.0) 0.05 M EDTA, 0.5% SDS). The glass rod was washed with an additional 40 *μ*L of extraction buffer and the homogenate was incubated at 65°C for 40 min. An amount of 10 *μ*L of 8 M potassium acetate was added and the tube was placed on ice for 40 min. The tube was spun at 14000 rpm for 20 min. The supernatant was transferred into a fresh 1.5 mL tube. One hundred microliters of chilled (−20°C) 100% ethanol was added and the DNA was allowed to precipitate at room temperature for 10 min. The tube was spun for 20 min and the ethanol was carefully removed with a pipette. The DNA pellet was washed with 100 *μ*L of chilled 70% ethanol and spun for 10 min. The DNA pellets were dried by pouring off the ethanol. The tubes were kept for 10 min at room temperature. The dried DNA pellet was suspended in 50 *μ*L TE (Tris EDTA, pH 8.0) and gently mixed for 10 min. The DNA concentration was measured using LKB-Ultrastep III UV/visible spectrophotometer at the absorbance of 260 nm and 280 nm. The DNA was considered pure if the ratio of OD_260_/OD_280_ was within the range of 1.6–1.9 [12].

### 2.4. Primers Used in This Study

A total of three forward sequencing primer denoted as DALP231, DALP234, DALP235 and a universal reverse primer, DALPR (5′TTTCACACAGGAAACAGCTATGAC-3′), were used for the PCR amplification (Table 2). Primer DALPR was end labeled with Y^33^ PATP (10 m Cie/mL) [14].

### 2.5. PCR Protocols

The PCR reaction mixture contained 60 ng of insect DNA, 1.8 mM of Mg^+^, 0.15 *μ*M of oligonucletide primer, 200 *μ*M of each dNTP, 1 unit Taq DNA polymerase (Promega), and 1x PCR buffer in a total volume of 25 *μ*L. Amplification reactions were carried out in a programmable thermal cycler (GeneAmp, PCR system 2400, Perkin Elmer) programmed as follows: predenaturation at 95°C for 2 min; followed by 30 cycles of denaturation at 91°C for 30 sec, annealing temperature at 55°C for 45 sec, and extension at 70°C for 30 sec. After the last cycle, final extension was at 70°C for 5 min. The protocols were modified from [8].

### 2.6. Electrophoresis of the Multilocus Amplification Products

Electrophoresis was performed on 6% denaturing polyacrylamide gels and run on a 50 cm long gel apparatus. The samples were mixed with 5 *μ*L 100% formamide loading dye and then heated for 10 min at 96°C before loading. The gel was run at 55 W for 3 hours.

### 2.7. Autoradiography

After electrophoresis, the gel was transferred to a Whatman paper and dried and developed after 5 days of exposures to X-ray film.

### 2.8. Statistical Analysis

**Band Scoring**

DALP-PCR band profiles were scored visually for each DNA sample for each primer pair. The data was recorded according to the presence/absence criterion (1 = presence; 0 = absence of band).

**Cluster Analysis**

The Dice algorithm was used for similarity index. The similarity index was calculated between two samples from within or between populations according to [15]:

(1) $$S_{xy}=\frac{2m_{xy}}{\left(m_{x}+m_{y}\right)},$$

where *m*_*xy*_ is the number of bands showed by sample *x* and sample *y* and *m*_*x*_ and *m*_*y*_ are the number of bands in sample *x* and sample *y*, respectively. The value produced by this index ranges from 0 (representing no band sharing) to 1 (representing complete identity). The within or between population values are based on pairwise comparisons between individuals for a particular primer. The values obtained are then averaged over primers.

The between population similarity indices were also converted to distance values using the relationship *D* = 1 − *S* [16, 17]. These distance matrices were used as the input matrix for the unweighted pair group method with arithmetic mean (UPGMA) tree [18] to find population relationships graphically using NTSYS-PC software (version 1.8; [19]).

**Test of Robustness**

The test of robustness or bootstrapping was performed using the Phylogeny Inference Package (PHYLIP; version 3.5p) developed by Felsenstein [20]. The bootstrap values were obtained using gene frequencies option within the program PHYLIP. A consensus tree was produced based on the 1000 bootstrapped replicates as reported by Haymer et al. [21].

**Principle Component Analysis**

A principal component analysis was performed based on the distance matrix among the populations using the NTSYS-PC software. The relationship among the populations is expressed in a three-dimensional graph based on the first three components.

**Analysis of Molecular Variance (AMOVA)**

The distance between two samples was calculated according to the formula of Excoffier et al. [22]:

(2) $$D=N\left\{1-\left(\frac{N_{xy}}{N}\right)\right\},$$

where *N* is the total number of bands and *N*_*xy*_ is the number of bands shared by two samples. The resulting distance matrix was used in an AMOVA [22]. In the AMOVA, the sources of variation were divided into three nested levels: among the host types, among the populations within host types, and among individuals within populations. Mean square deviation was calculated by dividing sum of squared deviation by the degrees of freedom. The variance component was expressed as percentage. The significance of components of variance was tested by the random permutation.

## 3. Results

**Figure 1:**
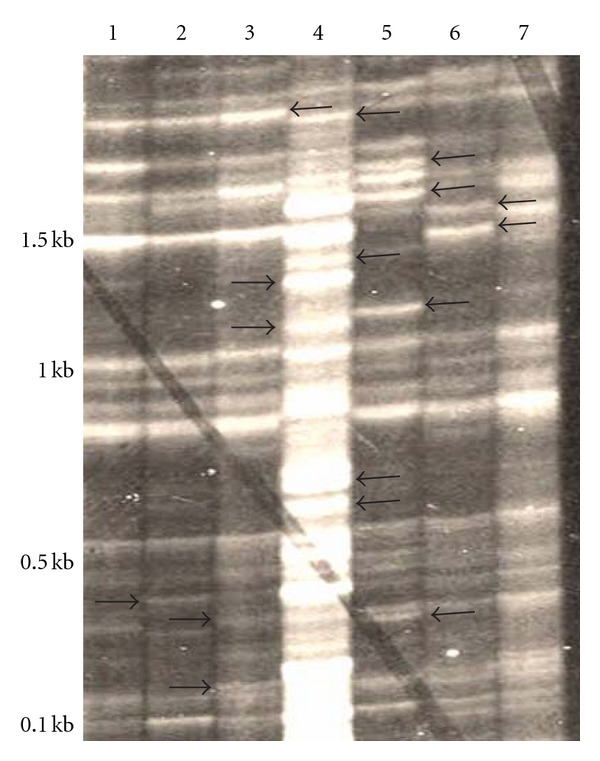
DALP-PCR amplicons obtained from rice and *Leersia* infesting populations of *N. lugens* using primer DALP235/DALPR (lanes 1–3 = rice infesting populations, lanes 5–7 = *Leersia* infesting populations; lane 4 = An out group, *N. bakeri*). Polymorphic markers showed in arrow sign.

Band or marker frequency was calculated for each marker pair for each population for DALP primers.  Figure 1 shows the banding patterns obtained from rice and *Leersia* infesting populations of *N. lugens* using primer DALP235/DALPR. A hundred percent marker frequency represented monomorphism while 0% showed complete absence of the particular marker. The data showed a range of 28.3–42.9% polymorphic markers for rice infesting populations of *N. lugens* while *Leersia* infesting populations and an out group,* N. bakeri* showed 31.9–45.5% and 17.1% polymorphic markers, respectively. The overall data for 10 populations of *N. lugens* based on three primers showed 29 (42.6%) polymorphic markers. Frequency of DALP markers, total number of markers, number of polymorphic markers, % polymorphic markers for each population are shown in Table 3.

### 3.1. Cluster Analysis

All data from three pairs of DALP primers were incorporated for cluster analysis. In this analysis, pairwise genetic distances were calculated between all individuals in order to make comparison. The distances within rice infesting populations of *N. lugens* ranged from 0.112487 to 0.285200 (average 0.2245843) while distances within *Leersia* infesting populations ranged from 0.152379 to 0.235396 (average 0.2078274). The genetic distances between two sympatric populations of *N. lugens*, one from rice and the other from grass, ranged from 0.24019 to 0.390182 (average 0.31672).

In addition to that genetic distances between rice infesting population of *N. lugens* and *Leersia *infesting populations of *N. bakeri* (out group) ranged from 0.555389 to 0.564963 (average 0.540276) but it was ranged from 0.499403 to 0.578171 (average 0.539168) between the populations of *Leersia* infesting *N. lugens* and *N. bakeri* (Table 4).

**Figure 2:**
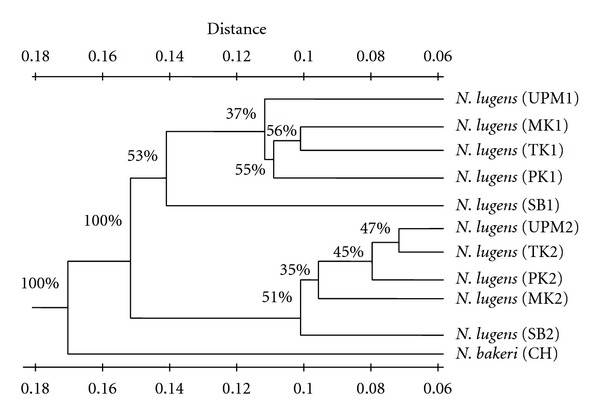
UPGMA dendrogram of the 11 populations of *Nilaparvata* spp. based on genetic distance from Dice's index for DALP markers (Rice infesting population of *N. lugens* = UPM1, TK1, MK1, PK1, and SB1; *Leersia*-infesting population of *N. lugens* = UPM2, TK2, MK2, PK2, and SB2; an out group, *N. bakeri* = CH); bootstrap values from 1000 bootstraps are given at each fork.

UPGMA dendrogram revealed the genetic relationships among the 11 populations of *Nilaparvata* species. The cluster analysis divided the individuals into three main clusters. Among the three clusters, one was the most distinct and distant and the other two were closely related. All rice infesting populations like UPM1, MK1, TK1, PK1, and SB1 were included in one cluster, likewise the *Leersia* infesting populations such as UPM2, TK2, PK2, MK2, and SB2 were separated into another group. A common branch was shared by both groups. The isolated population CH (*N. bakeri*) was far away from either rice or *Leersia* infesting populations of *N. lugens* (Figure 2).

### 3.2. Test of Robustness

The UPGMA tree was subjected to numerical resampling by bootstrapping [23] and the resultant bootstrap values were shown at the tree branch points. Each value represents the number of times that the represented groupings occurred in the resamplings. The consensus tree showed 100% confidence levels between rice (MK1, UPM1, TK1, PK1, and SB1) and *Leersia* infesting (MK2, TK2, UPM2, SB2, and PK2) population. Within rice and *Leersia* infesting populations, confidence level ranged from 37–56% to 35–51%, respectively (Figure 2). The confidence level between *N. lugens* and *N. bakeri* was also 100%.

### 3.3. Principal Component Analysis (PCA)

**Figure 3:**
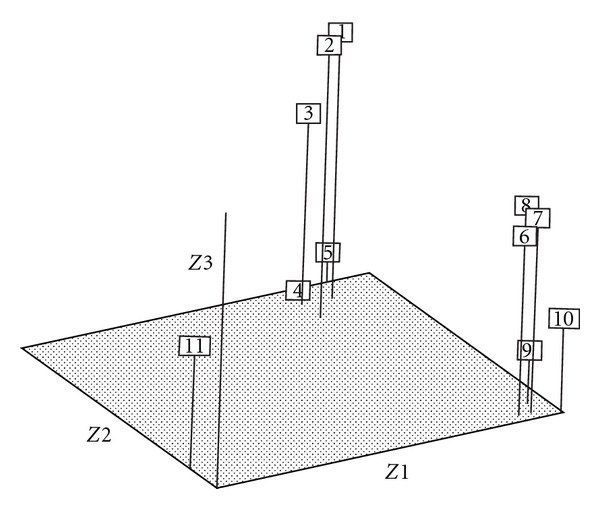
Patterns of relationships of the 11 populations revealed by the principal component analysis based on short primer DALP data. Proportion of the total variance explained by the first three principal components (PCs) is 78.31%: PC1 = 41.19%; PC2 = 27.42%; PC3 = 9.70% (Rice infesting populations of *N. lugens*: 1 = UPM1, 2 = TK1, 3 = MK1, 4 = PK1 and 5 = SB1; Grass infesting populations of *N. lugens*: 6 = UPM2, 7 = TK2, 8 = MK2, 9 = PK2, and 10 = SB2; An out group, *N. bakeri*, 11 = CH).

A principal component analysis was performed based on the distance matrix among the populations using the NTSYS-PC software. The relationship among the populations was expressed in a three-dimensional graph. In PCA graph, 11 populations were clustered into 3 groups. The cluster I consisted of rice infesting population of *N. lugens* while *Leersia* infesting populations showed another group. The population of *N. bakari* showed an out group. The first three principal components accounted for 78.31% of the total variation among the 11 populations of *Nilaparvata* spp. and these 3 components, PC1, PC2, and PC3 showed 41.19, 27.42, 9.70% variation, respectively (Figure 3).

### 3.4. Analysis of Molecular Variance (AMOVA)

Three level nested structures for each pair of primer of DALP are shown in Table 5. All primers showed variance among the host types, among the populations, and among the individuals in a population. Out of three primers, DALP 235 determined the highest variance among the groups (rice versus *Leersia*) (26.90%), followed by DALP231 (10.10%), and DALP234 (9.68%). The percentage of the variance component among groups (rice versus *Leersia*) was greater than the percentage of the variance component among the populations detected by the three primers DALP235, DALP234 and DALP231. The results of AMOVA as well as dendrogram confirmed that genetic variation exists between the brown plant hopper of rice and *Leersia*. 

## 4. Discussion

Cluster and principal component analyses revealed the genetic relationships among the different populations of *Nilaparvata* species. Three major clusters were observed in the dendrogram as well as in the graph. The results showed that population of *N. bakeri* formed the most isolated cluster from populations of either rice or *Leersia* infesting populations of* N. lugens*. The rice infesting populations of UPM, Tanjung Karang, Melaka, Perak, and Sabah, Malaysia clustered together as a group. On the other hand, *Leersia *infesting populations of the same localities formed another distinct cluster.* Leersia* infesting populations with low esterase activities seem to be formed, a different structure from rice infesting populations of brown planthopper,* N. lugens*. These results were also confirmed by bootstrapping analysis as described by Felsenstein [23] and Latif et al. [6]. Bootstrapping was initially used to evaluate the accuracy of a tree obtained by the parsimony method and could increase the confidence level of the results obtained from the DALP assay. The results showed 100% confidence level for the separate clusterings between the rice and *Leersia* infesting populations of *N. lugens* and also for the genetically isolated group, *N. bakeri*.

In DALP fingerprinting method, we did not get any diagnostic markers between two sympatric populations of *N. lugens*. Saxena and Barrion [24] reported that karyoytpe, idiogram, nuclear organelles, chromosomes with nucleolus organizing region (site of RNA synthesis) showed clear differences between rice and *Leersia* infesting populations. Despite their morphological similarities, a distinct cytological incongruity and a certain degree of genetic isolation between the two populations were inferred. Species differentiation in early stages of a species formation may not be associated with substantial genetic change [25–27]. Many ecologists have accepted that the evolutionary processes are common in animals with specialized food habits [28, 29]. There was no distinct electrophoretic differentiation between *Lethe eurydice* L. and *Lethe appalacia* L., although the two species were found to be good species [27, 30]. Latif et al. [6] reported that the closely related sibling species in the *N. lugens* complex might have developed through insecticide exposures that were heavier in rice-infesting populations than in grass populations, through RAPD-PCR analysis.

 The genetic distance indicates the magnitude of genetic variation between populations. Genetic distance commonly ranged from nearly 0.01 for populations within species, 0.1 for different subspecies, and 1.0 for different species [31]. So, the genetic distances (average 0.31672) between rice infesting populations (high esterase activities) and *Leersia* infesting populations (low esterase activities) of brown planthoppers indicated that these sympatric populations represented two distinct but closely related biological species.

The results of AMOVA in single primer yielded highly significant variance among group (rice versus *Leersia)* and among population components. The total genetic variation, an average 15.56% was attributable to group divergence (Rice versus *Leersia*), 13.01% to population differences and 77.37% to individual differences within a population. The percentage of variance component among groups (rice versus *Leersia*) was larger than the percentage of variance component among populations for bands detected by three DALP primers and these were tested by random permutation. These results revealed that there was genetic differentiation between the brown planthopper of rice versus *Leersia* (two sympatric populations of *N. lugens*). AMOVA was performed and was confirmed the differentiation into two groups of *Aphid gossypii* [32], five groups of *Acorus gramineus* [33], and two groups of natural populations of the wild rice, *Oryza rufipogon* [34]. Therefore, our molecular data of DALP-PCR indicated that brown plant hopper (BPH) with high esterase activity usually captured from rice plant and those with low esterase activity, usually captured from* L. hexandra* in Malaysia, represent two distinct closely related species and supported previous results as reported by Latif et al. [6, 7, 35]. Although DALP molecular method is not new, but so far to our knowledge this study is the first to detect genetic polymorphism in rice brown planthopper complex using this method.

## Figures and Tables

**Table 1:** Host types, sites of collection, and coding for 11 populations of *Nilaparvata* spp.

Species	Locations	Host plants	Population code
*N. lugens*	Universiti Putra Malaysia (UPM), Selangor, Malaysia	Rice	UPM1
*N. lugens*	UPM, Selangor, Malaysia	*L. hexandra*	UPM2
*N. lugens*	Tanjung Karang (Tk), Selangor, Malaysia	Rice	TK1
*N. lugens*	Tanjung Karang (Tk), Selangor, Malaysia	*L. hexandra*	TK2
*N. lugens*	Malim, Melaka (Mk), Malaysia	Rice	MK1
*N. lugens*	Malim, Melaka(Mk), Malaysia	*L. hexandra*	MK2
*N. lugens*	Bander Seberang, Perak (Pk), Malaysia	Rice	PK1
*N. lugens*	Bander, Seberang, Perak (Pk), Malaysia	*L. hexandra*	PK2
*N. lugens*	Tuaran, Sabah (SB), Malaysia	Rice	SB1
*N. lugens*	Tuaran, Sabah (SB), Malaysia	*L. hexandra*	SB2
*N. bakeri*	Cameron Highlands (CH), Pahang, Malaysia	*L. hexandra*	CH

**Table 2:** Optimisation of DALP primers used for the PCR protocols.

Primers	Sequence	Mg+2 conc.	Taq polymerase used	Annealing temperature (°C)
DALP231F	5′-GTTTTCCCAGTCACGACAGC-3′	1.8 mM	Promega	55
DALP234F	5′-GTTTTCCCAGTCACGACCAG-3′	1.8 mM	Promega	55
DALP235F	5′-GTTTTCCCAGTCACGACCAC-3′	1.8 mM	Promega	55
Universal DALPR	5′-TTTCACACAGGAAACAGCTATGAC-3′			

**Table 3:** Frequency of presence of bands (%) markers in *Nilaparvata* spp. obtained from three DALP primers.

Primer	Marker (M. Wt.)	UPM		Tanjung Karang		Melaka		Perak		Sabah		Cameron High lands
		*N. lugens* (Rice)	*N. lugens (Leersia*)	*N. lugens* (Rice)	*N. lugens (Leersia*)	*N. lugens* (Rice)	*N.lugens (Leersia)*	*N. lugens* (Rice)	*N.lugens (Leersia)*	N. lugens (Rice)	*N.lugens (Leersia)*	*N.bakeri (Leersia)*
	DALP231.1 (2.15 kb)	10	10	0	0	10	20	10	0	45	10	0
	DALP231.2 (2.0 kb)	0	25	0	0	40	30	15	0	50	10	30
	DALP231.3 (1.9 kb)	25	0	0	0	40	35	15	10	25	10	0
	DALP231.4 (1.8 kb)	100	65	100	90	100	90	100	80	70	100	80
	DALP231.5 (1.7 kb)	100	100	100	100	100	100	100	100	100	100	90
	DALP231.6 (1.6 kb)	100	100	100	100	100	100	100	100	100	100	0
	DALP231.7 (1.4 kb)	60	100	100	80	50	100	100	60	70	100	90
	DALP231.8 (1.3 kb)	100	100	100	100	100	100	100	100	100	100	100
	DALP231.9 (1.2 kb)	100	100	100	100	100	100	100	100	100	100	0
	DALP231.10 (1.0 kb)	0	60	0	50	0	40	0	60	0	40	100
DALP231	DALP231.11 (0.9 kb)	0	10	10	0	0	20	0	0	0	30	0
	DALP231.12 (0.85 kb)	30	40	25	50	30	30	20	30	40	35	80
	DALP231.13 (0.75 kb)	0	0	0	0	0	0	0	0	0	0	100
	DALP231.14 (0.70 kb)	0	0	0	0	0	0	0	0	0	0	100
	DALP231.15 (0.60 kb)	0	0	0	0	0	0	0	0	30	30	0
	DALP231.16 (0.50 kb)	0	0	0	0	0	0	0	0	0	0	100
	DALP231.17 (0.40 kb)	100	100	100	100	100	100	100	100	100	100	100
	DALP231.18 (0.30 kb)	100	100	100	100	100	100	100	100	100	100	0
	DALP231.19 (0.25 kb)	100	100	100	100	100	100	100	100	100	100	100
	DALP231.20 (0.20 kb)	80	60	100	100	100	100	100	100	100	100	0
	DALP231.21 (0.10 kb)	100	100	100	100	100	100	100	100	100	100	100
	DALP231.22 (0.09 kb)	0	0	0	0	0	0	0	0	0	0	100
	DALP234.1 (2.0 kb)	100	100	100	100	100	100	100	100	100	100	0
	DALP234.2 (1.9 kb)	100	100	100	100	100	100	100	100	100	100	0
	DALP234.3 (1.8 kb)	20	50	25	50	20	50	20	30	30	50	0
	DALP234.4 (1.75 kb)	0	0	0	0	0	0	0	0	0	0	100
	DALP234.5 (1.70 kb)	100	100	100	100	100	100	100	100	100	100	0
	DALP234.6 (1.65 kb)	0	70	0	40	0	40	0	60	0	40	100
DALP234	DALP234.7 (1.6 kb)	0	10	30	0	0	20	0	0	0	30	0
	DALP234.8 (1.5 kb)	40	40	60	50	30	30	20	30	40	35	100
	DALP234.9 (1.45 kb)	0	0	0	0	0	0	0	0	0	0	100
	DALP234.10 (1.3 kb)	100	100	100	100	100	100	100	100	100	100	100
	DALP234.11 (1.25 kb)	0	0	0	0	0	0	0	0	30	30	0
	DALP234.12 (1.10 kb)	0	0	0	0	0	0	0	0	0	0	100
	DALP234.13 (1.0 kb)	100	100	100	100	100	100	100	100	100	100	100
	DALP234.14 (0.9 kb)	100	100	100	100	100	100	100	100	100	100	0
	DALP234.15 (0.85 kb)	100	100	100	100	100	100	100	100	100	100	100
	DALP234.16 (0.7 kb)	100	100	100	100	100	100	100	100	100	100	0
	DALP234.17 (0.65 kb)	90	90	100	100	100	100	90	100	100	90	100
	DALP234.18 (0.5 kb)	0	0	0	0	0	0	0	0	0	0	100
	DALP234.19 (0.35 kb)	40	50	100	100	100	100	100	100	80	90	100
	DALP234.20 (0.30 kb)	80	20	80	70	60	40	50	90	70	80	100
	DALP234.21 (0.15 kb)	40	50	90	100	80	100	100	100	80	90	100
	DALP235.1 (2.2 kb)	5	10	10	8	0	10	9	20	40	35	0
	DALP235.2 (2.1 kb)	100	100	100	100	100	100	100	100	100	100	100
	DALP235.3 (2.0 kb)	100	100	100	100	100	100	100	100	100	100	0
	DALP235.4 (1.9 kb)	100	100	100	100	100	100	100	100	100	100	100
	DALP235.5 (1.8 kb)	10	40	15	50	20	35	16	30	10	50	0
	DALP235.6 (1.75 kb)	0	0	0	0	0	0	0	0	0	0	100
	DALP235.7 (1.70 kb)	100	100	100	100	100	100	100	100	100	100	0
	DALP235.8 (1.65 kb)	0	60	0	50	0	40	0	60	0	40	100
	DALP235.9 (1.6 kb)	0	10	10	0	0	20	0	0	0	30	0
	DALP235.10 (1.5 kb)	30	40	25	50	30	30	20	30	40	35	100
	DALP235.11 (1.4 kb)	0	0	0	0	0	0	0	0	0	0	100
	DALP235.12 (1.3 kb)	0	0	0	0	0	0	0	0	0	0	100
	DALP235.13 (1.2 kb)	0	0	0	0	0	0	0	0	30	30	0
	DALP235.14 (1.15 kb)	0	0	0	0	0	0	0	0	0	0	100
	DALP235.15 (1.0 kb)	100	100	100	100	100	100	100	100	100	100	100
DALP235	DALP235.16 (0.9 kb)	100	100	100	100	100	100	100	100	100	100	100
	DALP235.17 (0.8 kb)	100	100	100	100	100	100	100	100	100	100	100
	DALP235.18 (0.7 kb)	100	100	100	100	100	100	100	100	100	100	0
	DALP235.19 (0.6 kb)	100	100	100	100	100	100	100	100	100	100	100
	DALP235.20 (0.5 kb)	0	0	0	0	0	0	0	0	0	0	100
	DALP235.21 (0.35 kb)	30	50	100	100	100	100	100	100	80	90	70
	DALP235.22 (0.30 kb)	90	20	80	70	60	40	50	90	70	80	100
	DALP235.23 (0.20 kb)	60	90	50	30	80	100	90	60	40	75	100
	DALP235.24 (0.15 kb)	100	100	80	100	100	100	100	100	90	100	90
	DALP235.25 (0.10 kb)	100	100	100	100	100	100	100	100	100	100	100

Total Number of markers		45	51	46	46	45	52	46	47	49	55	41

Number of polymorphic markers		17	23	14	14	13	18	13	15	21	25	7

% Polymorphic markers		37.77	45.09	30.43	30.43	28.88	34.61	28.26	31.91	42.85	45.45	17.08

Overall number of markers based on the populations of *N. lugens *												68

Overall number of polymorphic markers based on the populations of *N. lugens *												29

Overall % poymorphism based on the populations of *N. lugens *												42.64

Frequency based upon 20 individuals per population.

**Table 4:** Genetic distance matrix of the 11 populations of *Nilaparvata* spp. based on Nei and Li's similarity index.

	UPM 1	TK1	MK1	PK1	SB1	UPM2	TK2	MK2	PK2	SB2	CH
UPM1	0										
TK1	0.112487	0									
MK1	0.169136	0.158045	0								
PK1	0.281682	0.285200	0.244057	0							
SB1	0.242807	0.250772	0.228062	0.273595	0						
UPM2	0.290265	0.292717	0.299515	0.372207	0.327829	0					
TK2	0.300214	0.285250	0.313183	0.378135	0.343346	0.152379	0				
MK2	0.240193	0.253426	0.273773	0.331707	0.284036	0.167285	0.183129	0			
PK2	0.306063	0.316361	0.311206	0.338466	0.326583	0.208954	0.222898	0.197168	0		
SB2	0.328876	0.332774	0.34628	0.390182	0.335617	0.235396	0.24859	0.228597	0.233878	0	
CH	0.555389	0.509043	0.497337	0.574649	0.564963	0.499403	0.525974	0.554652	0.537638	0.578171	0

UPM1, TK1, MK1, PK1, and SB1 are rice-infesting populations; UPM2, TK2, MK2, PK2, and SB2 are *Leersia*-infesting populations of *N. lugens*. CH is *N. bakeri*.

**Table 5:** Analysis of molecular variance (AMOVA) of 10 populations of *N. lugens* based on three DALP primers.

Primer	Source of variation	Degree of freedom	Sum of squared deviation	Mean squared deviation	Variance component	% Total	P*
DALP235/DALPR	Rice versus* Leersia* (Among groups)	1	19.50	16.81	0.159	26.90	<0.001
	Populations within group	8	22.34	2.87	0.172	26.29	<0.001
	Individuals within population	190	49.50	0.29	0.280	52.03	<0.001

DALP231/DALPR	Rice versus *Leersia *(Among groups)	1	9.58	7.58	0.075	10.10	<0.001
	Populations within group	8	9.99	2.00	0.041	6.98	<0.001
	Individuals within population	190	75.43	0.48	0.474	85.06	<0.001

DALP234/DALPR	Rice versus *Leersia* (Among groups)	1	3.04	3.09	0.034	9.68	0.031
	Populations within group	8	7.07	0.82	0.03	5.78	0.006
	Individuals within population	190	66.90	0.35	0.38	95.02	<0.001

*After 1000 random permutations; *P* = Probability level.
